# Synchronization of Human Autonomic Nervous System Rhythms with Geomagnetic Activity in Human Subjects

**DOI:** 10.3390/ijerph14070770

**Published:** 2017-07-13

**Authors:** Rollin McCraty, Mike Atkinson, Viktor Stolc, Abdullah A. Alabdulgader, Alfonsas Vainoras, Minvydas Ragulskis

**Affiliations:** 1 HeartMath Institute, Boulder Creek, CA 95006, USA; mike@heartmath.org; 2 NASA Ames Research Center, Moffett Field, CA 94035, USA; viktor.stolc-1@nasa.gov; 3 Director of Research and Scientific Bio-Computing, Prince Sultan Cardiac Center, Alhasa, Hofuf 31982, Saudi Arabia; kidsecho@yahoo.com; 4 Cardiology Institute, Lithuanian University of Health Sciences, Kaunas 44307, Lithuania; alfavain@gmail.com; 5 Department of Mathematical Modelling, Kaunas University of Technology, Kaunas 51368, Lithuania; minvydas.ragulskis@ktu.lt

**Keywords:** heliobiology, geomagnetic field, HRV, Schumann resonance, heart rate variability, solar wind, ANS, autonomic nervous system, cosmic rays, solar radio flux

## Abstract

A coupling between geomagnetic activity and the human nervous system’s function was identified by virtue of continuous monitoring of heart rate variability (HRV) and the time-varying geomagnetic field over a 31-day period in a group of 10 individuals who went about their normal day-to-day lives. A time series correlation analysis identified a response of the group’s autonomic nervous systems to various dynamic changes in the solar, cosmic ray, and ambient magnetic field. Correlation coefficients and *p* values were calculated between the HRV variables and environmental measures during three distinct time periods of environmental activity. There were significant correlations between the group’s HRV and solar wind speed, Kp, Ap, solar radio flux, cosmic ray counts, Schumann resonance power, and the total variations in the magnetic field. In addition, the time series data were time synchronized and normalized, after which all circadian rhythms were removed. It was found that the participants’ HRV rhythms synchronized across the 31-day period at a period of approximately 2.5 days, even though all participants were in separate locations. Overall, this suggests that daily autonomic nervous system activity not only responds to changes in solar and geomagnetic activity, but is synchronized with the time-varying magnetic fields associated with geomagnetic field-line resonances and Schumann resonances.

## 1. Introduction

All biological systems on Earth are exposed to an external and internal environment of fluctuating invisible magnetic fields of a wide range of frequencies [1]. These fields can affect virtually every cell and circuit to a greater or lesser degree. Numerous physiological rhythms have been shown to be synchronized with solar and geomagnetic activity [2,3,4,5,6].

Human regulatory systems are designed to adapt to daily and seasonal climatic and geomagnetic variations; however, sharp changes in solar and geomagnetic activity and geomagnetic storms can stress these regulatory systems, resulting in alterations in melatonin/serotonin balance [7,8,9], blood pressure, immune system, reproductive, cardiac, and neurological processes [10,11,12,13]. Disturbed geomagnetic activity is associated with the intensification of existing diseases, significant increases in myocardial infarction incidence and death, changes in blood flow, aggregation, and coagulation, increased blood pressure, cardiac arrhythmias, and seizures in epileptics [2,3,11,14,15,16,17,18,19,20,21,22].

During periods of increased solar activity, which peaks every 10.5 to 11 years, the sun emits increased ultraviolet (UV) energy and solar radio flux, which is measured by the 2.8 GHz signal (F10.7) [23,24]. Although the details of the physiological mechanisms in humans and animals are not yet fully understood, it is apparent that solar and magnetic influences affect a wide range of human health and behavioral processes, with the cardiovascular and nervous systems being the most clearly affected [4,25].

The application of heart rate variability (HRV) as an indicator of autonomic nervous system (ANS) function and dynamics has greatly increased in recent years in both clinical and research settings [26,27,28,29]. HRV is the naturally occurring change in the time intervals between adjacent pairs of heartbeats, and reflects the functional status of interdependent regulatory systems that operate on different time scales to adapt to environmental and psychological challenges [30]. Low levels of age-adjusted HRV indicate chronic stress, pathology, or inadequate functioning in various levels of regulatory control systems in the neuro axis, and is predictive of all-cause mortality [29,31,32]. Healthy levels of HRV indicate psychological resiliency, behavioral flexibility and capacity to effectively self-regulate and to adapt to changing social or environmental demands [26,33,34], one’s sense of coherence [35], the personality character traits of Self-Directedness [36], and performance on cognitive performance tasks requiring the use of executive functions [29].

A number of studies have found significant associations between magnetic storms and decreased HRV, suggesting that the cardiovascular system is impacted by geomagnetic disturbances [13,14,37,38,39,40,41,42,43,44]. Several of these studies found a ~25% reduction in the very low frequency (VLF) rhythm [39,40,41,45], which is most strongly associated with increased health risk [46]. The low frequency (LF) rhythms were also significantly reduced, while the high frequency (HF) rhythms were not. Dimitrova et al., found that during geomagnetic storms, both LF and HF measures as well as the ratio between low and high frequencies tended to be reduced [38].

Several early studies observed an “anticipatory reaction” in physiological measures that can occur 2 to 3 days prior to the start of magnetic storms. There were significant changes in heart rate, HRV, blood pressure, skin conductance, and subjective physiological complaints [6,38,47,48,49,50,51,52]. This anticipatory affect was first observed by Chizhevsky in the 1920s, prior to the knowledge of high frequency emissions such as X-rays and the gigahertz frequencies (solar radio flux) radiated by the sun. He suggested that some unknown radiation from the sun was responsible for this anticipatory effect [48]. Increased radiations produced by coronal ejections reach the earth in 8 min, while the increased density and speed of the solar wind takes several days to reach the Earth’s magnetosphere, resulting in a magnetic storm, which explains the early observations of an anticipatory effect.

Stoupel, et al., correlated increased geomagnetic activity combined with high levels of cosmic rays with increases in the number of emergencies and deaths during these periods, including increases in sudden cardiac death and cerebral strokes [53,54].

Considerably less attention has been given to potential links between ultra low frequency (ULF) waves and health or physiological functions. The most common source of ultra low frequency waves are field-line resonances, which exhibit the largest amplitudes of the magnetic waves occurring in the magnetosphere [55]. The frequency of these waves depends on the length of the magnetic field lines, the field strength, and the speed and density of the solar wind. Waves in the frequency range below 1 hertz are classified with respect to their waveform shape and frequency, where sinusoidal oscillations are called “Pc” (pulsations continuous) and irregular waveforms are defined as “Pi” oscillations (pulsations irregular). Each major type is subdivided into frequency regions related to different phenomena. Standing wave field-line oscillations are associated with Pc3 to Pc5 waves in the frequency range between 1 mHz and 100 mHz (periods of 1000 to 10 s). Oscillations classified as Pc1 and 2 are traveling waves with frequencies up to 5 Hz, which are typically associated with geomagnetic sub-storms [56]. Studies have shown that an increase in field-line resonances can affect the human cardiovascular system, likely due to the Pc frequencies overlapping with the rhythms of the autonomic nervous and cardiovascular systems [57]. Khabarova and Dimitrova also found that the ULF waves between 2–10 mHz had the strongest correlation with increases in blood pressure (0.6) compared to geomagnetic measures (0.3) [6]. In addition, Zenchenko et al. reported that in two-thirds of their experiments, they found a synchronization between heart rhythms and the ultra-low frequency components (0.5 to 3.0 mHz) of the geomagnetic field [58].

In the late 1950s, Schumann and Koenig measured a set of frequencies consistent with the mathematical model predicting an earth-ionospheric cavity resonance [59]. The frequency of the first Schumann resonance (SR), as they are now named, is approximately 7.83 Hz, with a (day/night) variation of about ±0.5 Hz. The other SR frequencies are ~14, 20, 26, 33, 39, and 45 Hz, which closely overlap with human brainwaves, such as alpha (8–12 Hz), beta (12–30 Hz), and gamma (30–100 Hz). This similarity between the frequencies produced by the brain and the SRs and the tendency of the electroencephalogram rhythms to become synchronous with SR activity was first reported by Koenig [60]. Pobachenko et al. [61] monitored the SRs and EEGs of 15 individuals over a six week period, and found that variations in the EEG were correlated with changes in the SR across the daily cycle, and the largest correlations between the EEGs and SRs were during periods of higher magnetic activity. Persinger et al. have also studied EEG activity and the SR in real-time, and demonstrated that several of the SR frequencies are clearly found in the spectral profiles of human brain activity [62,63]. In their studies, they found that the power within the EEG spectral profiles had repeated periods of coherence with the first three SR resonance frequencies (7–8 Hz, 13–14 Hz, and 19–20 Hz) in real-time. This suggests that changes in the SR parameters are related to changes in the solar wind, and that solar radiation can affect brain activity, including modulations in cognition and memory consolidation [63].

Here, we report the results of a study that examined the relationships between solar and geomagnetic activity and human nervous system function as reflected in HRV. This study is unique, because it examines changes in a one-month long, continuously recorded HRV data set of 10 participants compared to time-varying changes in solar, local geomagnetic, and Schumann resonance activity.

## 2. Methods and Procedures

### 2.1. Participants

Ten healthy individuals, 34 to 65 years old with a mean age of 53 years (2 males, 8 females) volunteered to participate in the study. Six were employees at the HeartMath Institute (HMI) located in Boulder Creek, CA, and four were recruited from the local community. All of the participants worked full- or part-time during daytime hours. Several of the HMI employees lived and worked remotely. One was located in southern CA (Palm Springs), one in the Monterey, CA area, and the remaining employees worked in two separate locations about 10 miles apart or in separate offices. One male participant dropped out after the first 3 days due to a skin irritation at the electrode sites; his data was not used in the analysis. The research met all applicable standards for the ethics of experimentation in accordance with the Declaration of Helsinki. Participants provided written informed consent prior to their participation in the study.

### 2.2. HRV Data Collection

All participants underwent daily 24-h ambulatory HRV recordings for 31 consecutive days between 6 September and 7 October 2011. Prior to the start of the study, each participant received instructions on attaching, starting, and stopping the recorders (Bodyguard, Firstbeat Technologies Ltd., Jyväskylä, Finland). They also received instruction on electrode placement and how to retrieve data from the recorder and upload it to the data collection FTP site. Firstbeat Uploader, a software utility created for uploading recorded data, was distributed to each participant via email to their home or work computer. Participants were instructed to stop the recorder each morning after waking up to start the day, and allowed them up to 50 min to shower or bathe before reattaching the recorder and starting the new day’s recording. Ambu Blue Sensor VL microporous breathable disposable electrodes were used for all of the recordings. The electrodes were placed in a modified V5 position. To minimize skin irritation over the 31 days, participants were encouraged to locate the electrodes around three different positions near the V5 electrode sites. The HRV recorder calculates the RR Interval (R is a point corresponding to the peak of the QRS complex of the ECG wave; and RR is the **interval** between successive Rs) from the electrocardiogram sampled at 1000 Hz. The RR interval data were stored locally in the device memory, and downloaded to a computer workstation once per week. Of the planned 279 (9 participants × 31 days) daily recordings, 91% (253) were collected.

### 2.3. HRV Measures

HRV is a physiological measure that reflects autonomic nervous system activity and dynamics. The HRV measures assessed were the inter-beat-interval (IBI), SDNN index (SNDNi), total power (TP), very low frequency (VLF), low frequency (LF), and high frequency (HF) power, and the LF/HF ratio. The international HRV Task Force Report on HRV divides heart rhythm oscillations into primary frequency bands: high frequency (HF), low frequency (LF), and very low frequency (VLF) [64]. The HF range is from 0.15 Hz to 0.4 Hz (rhythms occurring between 2.5 and 7 s), and reflects parasympathetic or vagal activity. The LF range is between 0.04 and 0.15 Hz (periods occurring between 7 and 25 s), which primarily reflects vagally mediated baroreceptor activity [65]. The VLF is the range between 0.0033 and 0.04 Hz (25 and 300 s). Low VLF power has stronger associations with all-cause mortality than the vagally mediated bands [32,46,66,67]. Experimental evidence suggests that this rhythm is intrinsically generated by the heart, and that the amplitude and frequency of this rhythm is modulated by efferent sympathetic activity associated with physical activity [68] or stress, and that sympathetic activations can cause its frequency to move up into the LF band during ambulatory monitoring [69]. Oscillations or events in the heart rhythm with a period of 5 min or longer are reflected in the VLF region of the spectrum, and can only be assessed with 24-h or longer recordings.

All of the HRV recordings were downloaded from the FTP site to a computer workstation and analyzed using DADiSP 2002. Inter-Beat-Intervals greater or less than 30% of the mean of the previous four intervals were considered artifacts, and were removed from the analysis record. Following an automated editing procedure, all of the recordings were manually reviewed by an experienced technician, and, if needed, corrected. Daily recordings were processed in consecutive 5-min segments in accordance with the standards established by the HRV Task Force [70]. Any 5-min segment with >10% of the IBIs either missing or removed in editing were excluded from the analysis. The mean of the inter-beat-intervals (IBI) was calculated for every hour in the recording. The results of the 5-min segments were averaged into hourly segments to match the time resolution of the Omni 2 data set measures. The local time stamps in the HRV recordings were converted to Coordinated Universal Time (UTC) to enable synchronization to the Omni 2 and other environmental data sets.

### 2.4. Environmental Measures

Space weather and environmental measures were obtained from three sources, comprising six measures. The solar wind speed (SWS), Kp index, Ap index, sunspot number, F10.7 index, and index polar cap north (PC(N)) were downloaded from NASA’s Goddard Space Flight Center’s Space Physics Data Facility as part of the Omni 2 data set. Cosmic ray (CR) counts were downloaded from the University of Oulu’s Sodankyla Geophysical Observatory’s website in Finland. Time varying magnetic field data were obtained from the HeartMath Institute’s global network of magnetometers, called the Global Coherence Monitoring System (GCMS) [71]. Data for this study were obtained from the site located in Boulder Creek, California. Three magnetic field detectors (Zonge Engineering ANT-4) are positioned in the north-south, east-west, and vertical axis to detect local time-varying magnetic field strengths (sensitivity 10^−12^ T) over a relatively wide frequency range (0.01–300 Hz) while maintaining a flat frequency response. The data acquisition infrastructure captures, stamps with global positioning system time, and transmits the data to a common server. Each magnetometer is continuously sampled at a rate of 130 Hz. The total hourly magnetic field variance (TMFV) is reflected by the standard deviation of the time-varying magnetic field (0.2 mHz–50 Hz) and reflects a direct measure of the activity in the magnetic field data, although the majority of the power is due to magnetic field-line resonances and ULF rhythms. Schumann resonance power (SRP) (0.32–36 Hz) was calculated as the average power spectral density for each 30 s, non-overlapping segment in each hour.

**Figure 1:**
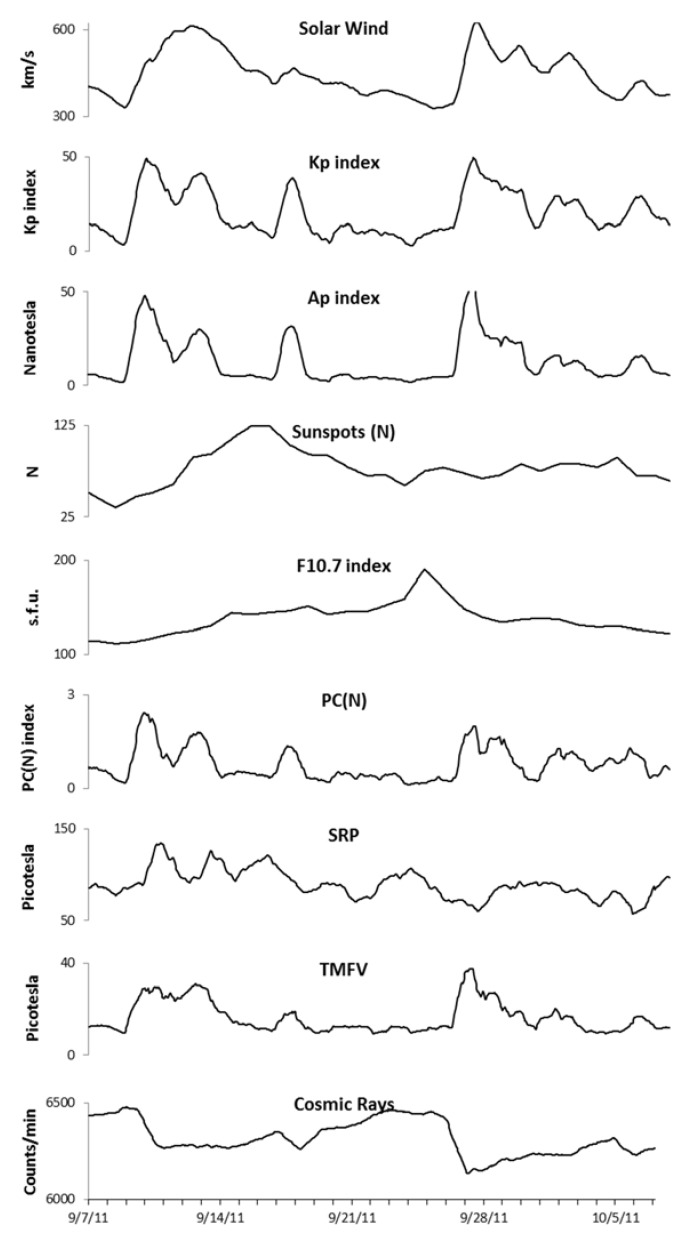
Shows changes in all the environmental data collected during the study period. TMFV, total hourly magnetic field variance; SRP, Schumann resonance power; PC(N), polar cap north.

The environmental data for the study period is shown in Figure 1. On 8 September an M-class solar flare occurred, which remained active for 3 days. This was followed by an increase in the SWS and the National Oceanic and Atmospheric Administration (NOAA) Kp index jumped from 2 on 8 September to 7 on 9 September, indicating a strong magnetic storm had begun. This is also reflected in the sharp increase in the Ap index and TMFV data on shown in Figure 1. The Kp index remained elevated through to 13 September, before settling down to normal levels. Then, another coronal mass ejection (CME) occurred on 14 September, which resulted in a moderate magnetic storm starting on 17 September (NOAA Kp 6). The field settled down to low activity levels until an X1.9 category CME occurred on 24 September, which included an extreme ultraviolet flash. This resulted in a sharp increase in SWS and a severe magnetic storm (Kp 8) starting on 26 September, with aftershocks keeping the magnetic field active or unsettled until 3 October.

### 2.5. Statistical Analysis

The participants’ time series data were time synchronized (hours with missing data were left empty). Each participant’s time series HRV data were then normalized to have a zero mean and amplitude that varied between −1 and 1. The mean of the participants’ time series was determined by taking the mean at each hourly time point. This method was used to generate a group average time series for each of the HRV measures.

Due to the small number of participants, the question arises as to whether the average HRV time series is representative of a true population average. Several statistical approaches were applied in order to gain more information about the resulting time series.

First, we plotted each of the group average HRV time series with a 95% confidence interval calculated from the standard error for each hourly data point over the 31-day period. As expected, the average IBI time series contains a clear circadian rhythm. All of the other group average HRV time series measures also had a circadian rhythm; however, the amplitude was smaller as compared to the IBIs.

Next, we bootstrapped over a single hourly time step (from each of the nine participants) to look at the distribution of the mean. Bootstrapping is a statistical resampling technique that was used to examine the distribution of the average HRV measures at different isolated points in time. A distribution of 1000 randomly sampled means was created from the original nine data points for a given HRV measure at a single point in time. This was done by calculating the mean of a randomly chosen set of nine new points from the original nine, allowing the possibility of individual data points being chosen more than once within a sample data set, and repeating the process 1000 times. This process was repeated for each HRV measure at 50 different arbitrarily selected time points. In all cases, it was found that the shapes of the distributions were normal, and our original data means were within one standard deviation of the bootstrap population mean. These results suggest that there is a relationship between the nine participants, at all of the data points tested, since unrelated data would have a distribution resembling noise.

Next, we analyzed the averaged HRV indices with the circadian rhythms removed. To remove the circadian rhythm from each of the time series, we used a centered 25-h moving average. The moving average adjusted the divisor if any missing data were within the averaging window. Missing data points were not interpolated.

The same 25-point moving average was also applied to the environmental time series.

For each average HRV measure, we bootstrapped without replacement to determine if any one participant was dominating the time series. This was determined by averaging 8 out of the 9 participants, applying the 25-point moving average, and correlating that new time series with the 9-participant average. The results showed that no one participant had a large effect on the resulting averages for any of the HRV measures (Table 1).

These statistical methods confirm that the HRV averages represent a relationship between the nine participants that is representative of the population average based on bootstrapping, and that no one participant had a dominant effect on these average time series.

In the following, the term averaged HRV time series refers to the nine participants’ averaged HRV rhythms with the circadian component removed.

## 3. Results

In the first analysis, correlation coefficients and P values were calculated for the averaged HRV time series and environmental measures. Correlations among the HRV measures are presented in Table 2. Correlations among the environmental measures are presented in Table 3, and are in agreement with previously established relationships [72,73,74].

Due to the number, strength, and type of CMEs that occurred during the recording period, it was evident that there were three different periods of environmental activity, each with distinct dynamics and interactions. We therefore divided the study period into three segments. During the first 14 days (unsettled period), there were several magnetic field disturbances as described above. Following a 4-day period of low activity, the severe storm (storm period) and aftershocks with intense UV activity occurred (Kp 8). During the third segment (post storm period), the field disturbances had subsided (Kp 2).

As shown in Table 4, a number of significant correlations were found between the HRV measures that reflect ANS activity and the environmental measures during each of the three periods. Here, we only describe correlations between key variables that had correlations of 0.3 and above (*p* <0.001). In the two-week long unsettled period, there was a positive correlation between the SWS and IBIs, and a negative correlation with the LF/HF ratio. During the severe storm period, the IBIs became negatively correlated with SWS. During the post storm period, the SDNNi, the TP, and VLF, LF, and HF power were also negatively correlated with SWS, and the LF/HF ratio became positively correlated.

The Kp, Ap, and PC(N) indexes had fewer correlations with the HRV variables across all three periods of environmental activity. During the *unsettled period*, the Ap index was negatively correlated with IBIs, and the Kp and PC(N) with the SDNNi. During the *post storm period*, the Ap index was negatively correlated with HF and LF power. During the *unsettled period*, the total magnetic field variation (TMFV) measure was negatively correlated with the SDNNi and LF power, and during the severe storm period the TMFV was positively correlated with the SDNNi, the TP, and VLF power. During the post storm period, the correlations with the TMFV switched back to negative with the SDNNi, the TP, and VLF, HF, and LF power.

During the unsettled period, the correlations with Schumann resonance power were weak and mostly non-significant. However, during the severe storm period, the correlations became strong and negatively correlated with the SDNNi, TP, VLF, HF, and LF power. These same findings carried over into the post storm period, where the LF/HF ratio additionally become positively correlated.

During the unsettled period, there was a negative correlation between cosmic ray counts and IBIs and a positive correlation with the LF/HF ratio. However, in the post storm period, the correlations became strong and positive with the IBIs, the SDNNi, the TP, and the VLF, HF, and LF power, while the LF/HF ratio was strongly negatively correlated.

During both the unsettled and severe storm periods the solar radio flux (F10.7) index was positively correlated with the SDNNi, the TP, and VLF, LF, and HF power, and negatively correlated with the LF/HF ratio. During the post storm period, the solar radio flux was reduced, and the correlations reversed and became negative with the SDNNi, the TP, and VLF, LF, and HF power.

**Figure 2:**
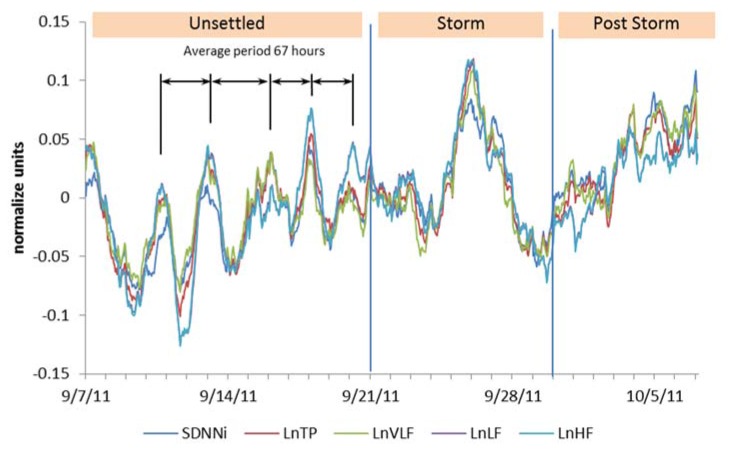
Slow wave HRV synchronization in the participants’ averaged SDNNi, TP, and VLF, LF, and HF power waveforms over the 30-day period.

An intriguing and surprising finding was the slow wave synchronization in the group averaged HRV data (SDNNi, TP, VLF, LF, and HF). A clear pattern of oscillations with an average period of 67 h (standard deviation (SD), 16.8 h) was observed during the first half of the study period (Figure 2). The arrows in the unsettled period indicate the varying time between the sine wave-like peaks in the oscillations of the slow wave HRV rhythm. This finding suggested that even though the participants were located at different locations, they were all synchronizing to external environmental signals with a similar oscillatory period.

**Figure 3:**
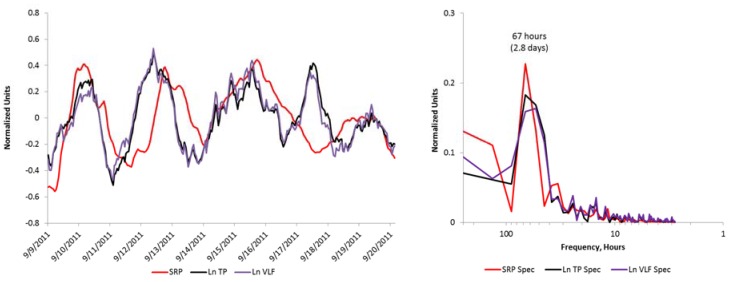
Overlay chart and frequency spectrum of the Schumann resonance power and the HRV metrics Total and VLF power.

To explore the data for the possibility that an external environmental signal may be influencing the HRV measures, we isolated the 11-day period with this clear oscillatory pattern, and examined the correlations between the SDNNi, the TP, and VLF, LF, and HF power waveforms and the environmental signals. Significant correlations were found between SRP and all five of the HRV measures. The SRP correlation with TP and VLF were the strongest (*r* = 0.46, *p* < 0.01 for both). An overlay chart and frequency spectrum of the three signals is shown in Figure 3, along with the frequency spectrum, which confirms that the primary peaks of these slow wave signals are oscillating at the same frequency.

**Figure 4:**
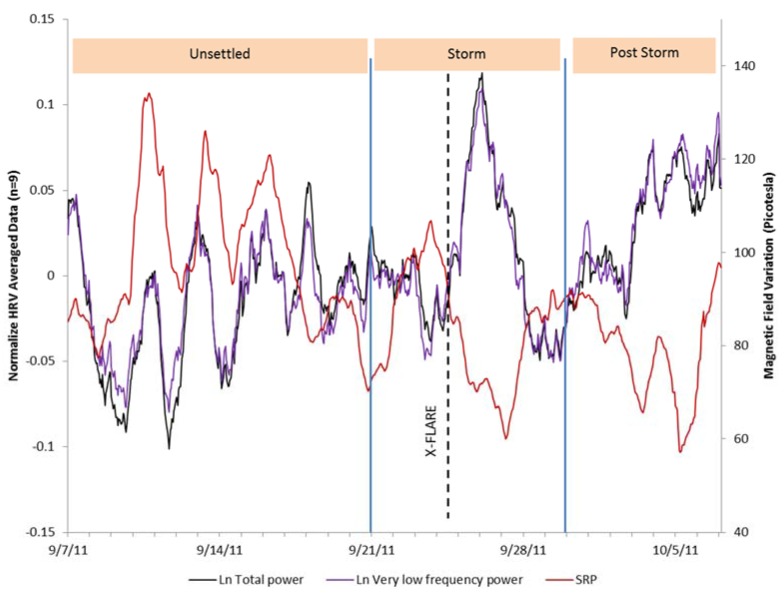
HRV total power and very low frequency power measures with the Schumann resonance power variations overlaid.

**Figure 5:**
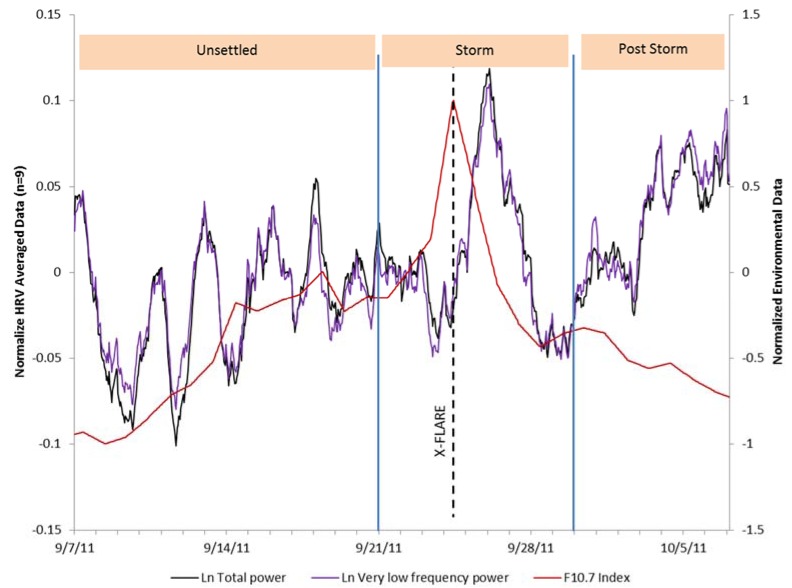
HRV total power and very low frequency power measures with the solar radio flux (F10.7) variations overlaid.

**Figure 6:**
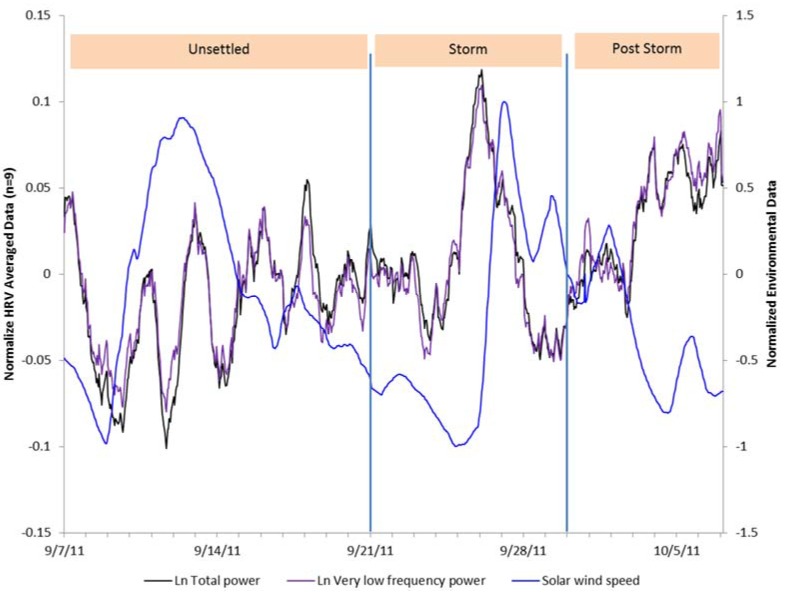
HRV total power and very low frequency power measures with solar wind speed variations overlaid.

Figure 4 shows an overlay of the HRV indices of VLF and total power and the Schumann resonance power across the 31-day period. The positive relationship can clearly be seen in the first 14 days of the study period up until the time of the strong X1.9 category CME that occurred on 24 September, which was accompanied by an extreme ultraviolet flash, X-ray burst, and a large peak in the solar radio flux (F10.7) index. During the magnetic storm and post-storm periods, the SRP dropped. The HRV during the storm appeared to be responding to other environmental factors during these periods. At the end of the post-storm period, the SRP began to recover. As can be seen in Figure 5, after the CME occurred there was an immediate increase in solar radio flux, which occurred at the same time as the X-class solar flare (dashed line), then declined over the post-storm period to nearly the same level as it was at the beginning of the study period. There was a steep increase in the group’s HRV following the increased solar radio flux, which rapidly declined in conjunction with the sharp jump in SWS on 26 September (Figure 6) and the beginning of the severe magnetic storm at approximately 12:15 universal time on 26 September (NOAA Kp index of 8). The Goddard Space Weather Lab reported a “strong compression of Earth’s magnetosphere”. Shortly after the start of the severe magnetic storm disturbance, the clear rhythm seen in the first two weeks of the SRP and the group’s HRV rhythm were disrupted. The positive relationship between the SRP and HRV became negative during the time of the disturbance, as discussed above. Once the magnetic field disturbance passed, the group’s HRV rhythms began to resynchronize in a similar pattern as the one seen in the first two weeks of the study period.

## 4. Discussion

This study was unique for several reasons: first, it utilized continuous monitoring of HRV over a 31-day period in a group of individuals that went about their normal day-to-day lives. Then, by chance, a number of dynamic changes in the solar, cosmic ray, and magnetic environment occurred that allowed the opportunity to examine how the group’s ANS responded to these various changes.

Overall, this study suggests that daily autonomic nervous system activity not only responds to changes in solar and geomagnetic activity, but also can synchronize with the time-varying magnetic fields associated with geomagnetic field-line resonances and Schumann resonances. The major influence that impacts these resonances and the earth’s magnetic field are the sun and solar wind [72,75]. Overall, the TMFV measure had much higher correlations with the HRV variables than the Kp, Ap, or PC(N) measures.

In regards to the HRV correlations, IBIs have an inverted relationship to heart rate (HR), with longer IBIs equating to a slower HR (HR = 60/IBI). HR and IBIs can be used as indicators of shifts in the relative balance between sympathetic and parasympathetic activity, and how the ANS responds or adapts to stress and challenges [26]. SWS was negatively correlated with IBIs during the magnetic storm period, indicating that HR increases along with increases in SWS and magnetic field disturbances, which can be interpreted as a physiological stress reaction with a carryover effect into the post-storm period, which is consistent with other studies [38,76].

The positive correlation between solar radio flux (F10.7) and cosmic rays for most of the HRV variables, along with the negative correlation between the LF/HF ratio during the first two weeks (unsettled period) of data collection, suggests that parasympathetic nervous system activity is enhanced during times of increased solar radio flux and cosmic rays. Decreases in the LF/HF ratio indicate higher parasympathetic activity relative to sympathetic activity in ambulatory HRV recordings [26]. This was an interesting finding, as a previous study also found that an increase in solar radio flux index was associated with lower fatigue, increased positive affect, and mental clarity, while increased SWS had the opposite effects [77]. These findings are also consistent with another study that took place over a 5-month period, which examined the time lags in ANS responses to changes in solar and magnetic variables [77]. In that study, HRV was also positively correlated with solar radio flux. After an initial increase in parasympathetic activity, the main effect started around 20 h after an increase in the F10.7, which is consistent with the finding in this study. The solar radio flux may, therefore, be one of the mediators of the anticipatory reactions first observed by Chizhevsky. Of course, other radiation sources such as X–rays, UV emissions, and cosmic rays from the sun emitted during coronal mass ejections are likely involved in the ANS reactions that occur prior to changes in SWS and geomagnetic disturbances.

In another long-term study that took place during a period of mostly magnetically quiet days, there were strong positive correlations between cosmic rays and HRV variables, suggesting a beneficial response to increases in cosmic rays [76]. Other studies have also suggested beneficial effects, at least in healthy populations. One such study examined serum C-reactive protein levels in a population suspected of having inflammatory-related problems, and found a strong and inverse correlation between C-reactive protein levels and cosmic rays [78]. In the first 2-week period in this study, the cosmic ray counts were weakly and negatively correlated to HRV measures, but had strong positive correlations with the LF/HF ratio measures. There was also a sharp increase in SWS and a strong reduction in CRs during the early part of this period in response to a moderate magnetic storm, which appeared to have an impact on HRV measures. During the post-severe storm period when CRs stared to increase as the solar wind decreased, there were strong positive correlations with CRs and negative SWS correlations on all the key HRV measures. This suggests that the CRs may have a stronger influence on ANS activity than the solar radio flux.

The mostly negative correlations between the SRP, IBIs, and HRV measures which occurred during the severe storm and post-storm segments is of interest, because the long-term study mentioned above found that SRP was strongly and positively associated with increased HRV across all measures and IBIs (lower heart rate). This suggests a beneficial effect of increased SRP, which was also supported by findings of reduced diastolic, systolic, and mean blood pressure during periods of higher SRP [79]. In addition, Persinger has conducted studies showing that the primary frequencies of the human brain are similar to SRs, and that real-time coherence between human brainwaves and the SRs can occur globally, with the power of the SRs being related to the degree of coherence [62,80]. The weak and negative correlations found in this study are likely due to several factors. The long-term study looked at the time lags in the ANS responses over a 40-h period following changes in environmental variables, and for changes in SRP the positive response lagged increases in SRP by 9 h [76].

Figure 3 shows an overlay of the SRP and HRV, and shows that the two are well-correlated with a clear oscillatory pattern during most of the first 2-week period, suggesting that environmental magnetic fields influence the autonomic nervous system as reflected by the HRV measures. Overall, during magnetically quieter periods, the SRP appears to play an important role in synchronizing people’s slow wave heart rhythms. The potential importance of these rhythms is currently unknown, but may be important to better understand human health and well-being.

It has been found that individuals have different degrees of sensitivity to the Earth’s magnetic fields, and can even respond in opposite ways to changes in the same environmental variable [6]. Collaborators at the Lithuanian University of Health Sciences have developed a method based on HRV for evaluating human sensitivity to local Earth magnetic field variations [81]. Future research should examine these slow wave dynamics in more detail.

### Limitations

The small sample size was the primary limitation of the study, which limits the surprising finding that the group’s heart rhythms were synchronized with each other. In addition, although the series of various classes of CMEs that occurred during the study period provided a unique opportunity to examine ANS responses to these events, it also complicated determining which environmental signal or signals the participants may be synchronizing to. As the participants were also located in a relatively small geographical area, although spread across the state of California, it was not possible to determine if the HRV synchronization could occur globally. In order to address these limitations, a second study has been conducted with over 100 participants with groups located in five widely distributed countries. A preliminary analysis of that study has confirmed that HRV synchronization occurs globally, and that the rhythms in SRP and ULF power appear to be the primary environmental factors that underlie group synchronization.

Although this study is inherently correlational due to the independent measures of interest, it is a limitation. However, the convergence of the results from other studies using different designs, sampling increments, populations, and geographic locations adds support to these findings. Although correlations, even strong ones, do not imply causation, correlations must occur if theories regarding causality are correct. Except in the rare case in which strong third-variable effects exist across studies, an absence of correlation between two variables indicates an absence of causality in either direction. Because a single study or type of evidence can never be definitive, an argument for causality can best be suggested when different types of evidence converge on the same conclusion. Therefore, we have discussed several types of evidence in order to test the hypothesis that daily autonomic nervous system activity, as reflected by HRV, reacts to changes in solar and geomagnetic activity in different ways depending on the dynamics occurring in the Earth’s magnetic and energetic environment, and that these reactions can persist over different lengths of time.

## 5. Conclusions

The results of this study are consistent with other studies showing that changes in solar and geomagnetic activity correlate with changes in human nervous system activity. Overall, the study suggests that daily autonomic nervous system activity not only responds to changes in solar and geomagnetic activity, but is synchronized with the time-varying magnetic fields associated with geomagnetic field-line resonances and Schumann resonances. A likely explanation for how solar and geomagnetic fields can influence human nervous system activity is through a resonant coupling between our nervous systems and geomagnetic frequencies (Alfvén waves), or ultra low frequency standing waves in the earth-ionosphere resonant cavity (Schumann resonances) that overlap with physiological rhythms.

## Figures and Tables

**Table 1:** HRV Bootstrap correlations for the nine participants average vs average excluding one participant.

	IBI	SDNNi	ln TP	ln VLF	ln LF	ln HF	ln LF/HF
Excluding 1	0.99	0.95	0.94	0.93	0.94	0.92	0.94
Excluding 2	0.99	0.98	0.97	0.98	0.94	0.94	0.97
Excluding 3	0.96	0.87	0.95	0.92	0.98	0.98	0.98
Excluding 4	0.98	0.98	0.94	0.93	0.96	0.99	0.98
Excluding 5	0.96	0.96	0.94	0.95	0.94	0.96	0.96
Excluding 6	0.93	0.99	0.97	0.97	0.93	0.97	0.98
Excluding 7	0.98	0.98	0.98	0.98	0.99	0.97	0.97
Excluding 8	0.95	0.97	0.97	0.96	0.97	0.96	0.97
Excluding 9	0.97	0.97	0.94	0.94	0.98	0.96	0.97

**Table 2:** HRV Measures—Correlation Coefficients.

	1	2	3	4	5	6	7
1. Mean IBI, ms	1						
2. SDNNi, ms	0.52 ^***^	1					
3. ln TP, ms^2^/Hz	0.53 ^***^	0.95 ^***^	1				
4. ln VLF, ms^2^/Hz	0.60 ^***^	0.94 ^***^	0.97 ^***^	1			
5. ln LF, ms^2^/Hz	0.31 ^**^	0.83 ^***^	0.90 ^***^	0.81 ^***^	1		
6. ln HF, ms^2^/Hz	0.50 ^***^	0.87 ^***^	0.91 ^***^	0.85 ^***^	0.83 ^***^	1	
7. ln LF/HF	−0.59 ^***^	−0.55 ^***^	−0.55 ^***^	−0.55 ^***^	−0.28 ^**^	−0.73 ^***^	1

^**^ *p* < 0.01, ^***^ *p* < 0.001.

**Table 3:** Environmental measures—correlation coefficients.

	Solar Wind	Kp Index	Ap Index	Sunspots	F10.7	PC(N)	SRP	TMFV	Cosmic Rays
Solar wind speed, Km/s	1								
Kp Index	0.73 ^***^	1							
Ap Index, nT	0.65 ^***^	0.94 ^***^	1						
Sunspots, n	0.18 ^***^	−0.08 ^*^	−0.13 ^***^	1					
F10.7, sfu	0.04	−0.15 ^***^	−0.09 ^**^	0.33 ^***^	1				
PC(N) index	0.54 ^***^	0.90 ^***^	0.87 ^***^	−0.16 ^***^	−0.33 ^***^	1			
SRP, pT	0.11 ^**^	−0.11 ^**^	−0.06	0.30 ^***^	−0.09 ^**^	−0.10 ^**^	1		
TMFV, pT	0.78 ^***^	0.87 ^***^	0.90 ^***^	−0.13 ^***^	−0.09 ^*^	0.78 ^***^	0.08 ^*^	1	
Cosmic Ray, counts/min	−0.72 ^***^	−0.54 ^***^	−0.42 ^***^	−0.24 ^***^	−0.04	−0.35 ^***^	0.13 ^***^	−0.45 ^***^	1

^*^ *p* < 0.5, ^**^ *p* < 0.01, ^***^ *p* < 0.001 Abbreviations: sfu, solar flux units, 1 sfu = 10^−22^ watt per square meter-Hz.

**Table 4:** Correlations between HRV and environmental measures.

**Unsettled Period**									
	**Solar Wind**	**Kp Index**	**Ap Index**	**Sunspots**	**F10.7 Index**	**PC(N)**	**SRP**	**TMFV**	**Cosmic Rays**
IBI, ms	0.38 ^***^	−0.15 ^**^	−0.32 ^***^	0.25 ^***^	0.09	−0.18 ^**^	0.28 ^***^	0.02	−0.39 ^***^
SDNNi, ms	−0.18 ^**^	−0.30 ^***^	−0.35 ^***^	0.41 ^***^	0.44 ^***^	−0.32 ^***^	−0.03	−0.42 ^***^	−0.15 ^**^
ln TP, ms^2^/Hz	−0.08	−0.14 ^**^	−0.21 ^***^	0.38 ^***^	0.42 ^***^	−0.19 ^***^	0.10	−0.28 ^***^	−0.23 ^***^
ln VLF, ms^2^/Hz	−0.02	−0.10	−0.17 ^**^	0.34 ^***^	0.28 ^***^	−0.12 ^*^	0.16 ^**^	−0.20 ^***^	−0.19 ^***^
ln LF, ms^2^/Hz	−0.19 ^***^	−0.14 ^**^	−0.18 ^**^	0.24 ^***^	0.41 ^***^	−0.19 ^***^	−0.04	−0.31 ^***^	−0.12 ^*^
ln HF, ms^2^/Hz	−0.17 ^**^	−0.13 ^*^	−0.17 ^**^	0.24 ^***^	0.41 ^***^	−0.18 ^***^	−0.05	−0.29 ^***^	−0.13 ^*^
ln LF/HF	−0.33 ^***^	0.15 ^**^	0.30 ^***^	−0.54 ^***^	−0.54 ^***^	0.26 ^***^	−0.07	0.16 ^**^	0.58 ^***^
**Storm Period**									
	**Solar Wind**	**Kp Index**	**Ap Index**	**Sunspots**	**F10.7 Index**	**PC(N)**	**SRP**	**TMFV**	**Cosmic Rays**
IBI, ms	−0.30 ^***^	−0.15 ^*^	−0.04	−0.01	0.64 ^***^	−0.13	0.20 ^**^	−0.06	0.28 ^***^
SDNNi, ms	−0.03	0.12	0.24 ^***^	−0.06	0.21 ^**^	0.15 ^*^	−0.69 ^***^	0.36 ^***^	0.13
ln TP, ms^2^/Hz	−0.09	0.11	0.22 ^**^	0.06	0.13 ^*^	0.13	−0.69 ^***^	0.34 ^***^	0.15 ^*^
ln VLF, ms^2^/Hz	−0.08	0.14 ^*^	0.26 ^***^	0.14 ^*^	0.18 ^**^	0.15 ^*^	−0.70 ^***^	0.36 ^***^	0.14 ^*^
ln LF, ms^2^/Hz	−0.20 ^**^	0.02	0.12	-0.01	0.22 ^**^	0.08	−0.59 ^***^	0.26 ^***^	0.23 ^***^
ln HF, ms^2^/Hz	−0.20 ^**^	0.02	0.13	0.00	0.23 ^***^	0.08	−0.59 ^***^	0.27 ^***^	0.23 ^***^
ln LF/HF	−0.40 ^***^	−0.23 ^***^	−0.24 ^***^	−0.23 ^***^	0.43 ^***^	−0.10	0.21 ^**^	−0.18 ^**^	0.26 ^***^
**Post-Storm Period**									
	**Solar Wind**	**Kp Index**	**Ap Index**	**Sunspots**	**F10.7 Index**	**PC(N)**	**SRP**	**TMFV**	**Cosmic Rays**
IBI, ms	−0.61 ^***^	0.09	−0.01	−0.40 ^***^	−0.79 ^***^	0.17 ^*^	−0.68 ^***^	−0.22 ^**^	0.53 ^***^
SDNNi, ms	−0.79 ^***^	−0.14	−0.25 ^**^	−0.47 ^***^	−0.80 ^***^	0.06	−0.32 ^***^	−0.32 ^***^	0.69 ^***^
ln TP, ms^2^/Hz	−0.80 ^***^	−0.14	−0.25 ^***^	−0.30 ^***^	−0.77 ^***^	0.15 ^*^	−0.46 ^***^	−0.34 ^***^	0.76 ^***^
ln VLF, ms^2^/Hz	−0.82 ^***^	−0.12	−0.19 ^*^	−0.46 ^***^	−0.80 ^***^	0.05	−0.44 ^***^	−0.30 ^***^	0.67 ^***^
ln LF, ms^2^/Hz	−0.79 ^***^	−0.26^***^	−0.37 ^***^	−0.31 ^***^	−0.87 ^***^	−0.06	−0.51 ^***^	−0.58 ^***^	0.63 ^***^
ln HF, ms^2^/Hz	−0.77 ^***^	−0.25^***^	−0.37 ^***^	−0.30 ^***^	−0.87 ^***^	−0.05	−0.51 ^***^	−0.58 ^***^	0.62 ^***^
ln LF/HF	0.42 ^***^	0.24^**^	0.21 ^**^	−0.08	0.10	−0.13	0.46 ^***^	0.25 ^**^	−0.63 ^***^

^*^ *p* < 0.05, ^**^ *p* < 0.01, ^***^ *p* < 0.001.

## Abbreviations

ANS	Autonomic nervous system
EEG	Electroencephalogram
HRV	Heart rate variability
HF	High frequency
LF	low frequency
LF/HF ratio	low frequency divided by high frequency power
VLF	very low frequency
SNDNi	SDNN index
TP	Total power
IBI	Inter-beat-interval
ULF	Ultra low frequency
Environmental related measures	
Pc	pulsations continuous
Pi	pulsations irregular
SR	Schumann resonance
SRP	Schumann resonance power
SWS	Solar wind speed
PC(N)	index polar cap north
CR	Cosmic rays
TMFV	Total magnetic field variance
CME	coronal mass ejection
